# Structure of Importin-α from a Filamentous Fungus in Complex with a Classical Nuclear Localization Signal

**DOI:** 10.1371/journal.pone.0128687

**Published:** 2015-06-19

**Authors:** Natalia E. Bernardes, Agnes A. S. Takeda, Thiago R. Dreyer, Fernanda Z. Freitas, Maria Célia Bertolini, Marcos R. M. Fontes

**Affiliations:** 1 Departamento de Física e Biofísica, Instituto de Biociências, Universidade Estadual Paulista, UNESP, Botucatu, SP, Brazil; 2 Departamento de Bioquímica e Tecnologia Química, Instituto de Química, Universidade Estadual Paulista, UNESP, Araraquara, SP, Brazil; Institute of Enzymology of the Hungarian Academy of Science, HUNGARY

## Abstract

*Neurospora crassa* is a filamentous fungus that has been extensively studied as a model organism for eukaryotic biology, providing fundamental insights into cellular processes such as cell signaling, growth and differentiation. To advance in the study of this multicellular organism, an understanding of the specific mechanisms for protein transport into the cell nucleus is essential. Importin-α (Imp-α) is the receptor for cargo proteins that contain specific nuclear localization signals (NLSs) that play a key role in the classical nuclear import pathway. Structures of Imp-α from different organisms (yeast, rice, mouse, and human) have been determined, revealing that this receptor possesses a conserved structural scaffold. However, recent studies have demonstrated that the Impα mechanism of action may vary significantly for different organisms or for different isoforms from the same organism. Therefore, structural, functional, and biophysical characterization of different Impα proteins is necessary to understand the selectivity of nuclear transport. Here, we determined the first crystal structure of an Impα from a filamentous fungus which is also the highest resolution Impα structure already solved to date (1.75 Å). In addition, we performed calorimetric analysis to determine the affinity and thermodynamic parameters of the interaction between Imp-α and the classical SV40 NLS peptide. The comparison of these data with previous studies on Impα proteins led us to demonstrate that *N. crassa* Imp-α possess specific features that are distinct from mammalian Imp-α but exhibit important similarities to rice Imp-α, particularly at the minor NLS binding site.

## Introduction

The filamentous fungus *Neurospora crassa* has been studied by classical and molecular genetics, providing several insights into cellular processes, which include cell signaling, growth and differentiation, secondary metabolism, circadian rhythm and genome defense [1]. Together with the yeasts *Saccharomyces cerevisiae* and *Schizosaccharomyces pombe*, *N. crassa* has largely been used as a model organism to study fundamental aspects of eukaryotic biology. Nevertheless, compared to yeast, *N. crassa* has developed greater morphological and developmental complexity due its multicellular characteristics, and although *N. crassa* is not known to be a pathogen, similarities exist between the saprotrophic Neurospora and pathogenic fungi [1].

The genomic sequence of *N. crassa* exhibits an apparent lack of functional gene duplication [1], which has allowed several studies to better understand the metabolic pathways of this experimentally tractable organism. We have used *N. crassa* as a model organism to understand the regulation of basic cellular processes, such as glycogen metabolism. Many proteins and transcription factors have been identified that likely play a role in this process [2, 3]. Some of these proteins and all transcription factors possess putative nuclear localization signals (NLSs), either monopartite or bipartite, suggesting that they can be transported into the nucleus via the classical nuclear pathway [3], which is mediated by importins (importin-*α*/importin-*β*) [4, 5].

The classical NLSs (cNLSs) are the best characterized targeting signals and are typically divided into one (monopartite) or two (bipartite) clusters of basic residues [6], whose consensus sequences correspond to K[K/R]X[K/R] for monopartite cNLSs and [K/R][K/R]X_10_12_[K/R]_3/5_ for bipartite cNLSs, where [K/R]_3/5_ corresponds to at least three of either lysine or arginine residues of five consecutive amino acids [6–8]. Structural studies have shown that both classes are recognized by the nuclear import receptor importin-*α* (Imp*α*) [7, 9, 10]. Imp*α* is composed of tandem armadillo (Arm) repeats that generate a curved and elongated shape. Imp*α* also contains conserved residues that form two NLS binding sites known as major (Arms 1–4) and minor (Arms 4–8) NLS binding sites [9, 10]. Monopartite NLSs primarily interact with the major binding site, whereas bipartite NLSs interact with both minor and major NLS binding sites [10–12].

The modulation of differential expression is a key feature for understanding the metabolism and growth of an organism [13, 14], and an understanding of the nuclear import mechanism of fungi may provide insights into these pathways. Although, Imp*α* structures have been elucidated, an understanding of the specificity of NLS recognition and regulation remains limited [15]. To better understand NLS recognition and gain insights into the *N. crassa* nuclear import process, we report the first crystal structure of Imp*α* from a filamentous fungus (NcImp*α*) in complex with a classical monopartite NLS (SV40 large T antigen NLS). This is also the highest resolution Imp*α* structure solved to date (1.75Å). Isothermal titration calorimetric (ITC) studies were used to determine the affinity and thermodynamic parameters of the interaction between Imp*α* and the classical SV40 NLS peptide. Both crystallographic and calorimetric analyses confirmed NcImp*α* recognition of cNLS via both major and minor binding sites. Moreover, we observed interactions in the minor binding site that are more similar to those in Imp*α* from rice (*Oryza sativa*) [16] than those in Imp*α* from yeast [9]. This study highlights the importance of the analysis of the interactions between NLSs and Imp*α* proteins from various organisms. Particularly, it may aid in the identification of specific NLSs from *N. crassa*, providing insights into the nuclear import pathway in fungi and enabling future biotechnological applications.

## Materials and Methods

### Protein expression and purification

The recombinant Imp*α* protein from *N. crassa* (NcImp*α*) was expressed as a truncated hexa-His fusion protein, consisting of residues 75–529, using the *Escherichia coli* host strain RosettaTM (DE3) pLysS (Novagen), as previously described [17]. The protein was purified using nickel affinity chromatography and eluted in a 0.15–3.0 M imidazole linear gradient, followed by dialysis. NcImp*α* was stored under cryogenic temperatures in a buffer composed of 20 mM Tris-HCl, pH 8, and 100 mM NaCl.

### Synthesis of NLS peptides

The peptide corresponding to SV40 NLS (^125^ *PPKKKRKV* ^132^) was synthesized by Proteimax (Brazil) with a purity of higher than 99%. The peptides contained additional residues at the N- and C-termini compared with the minimally identified NLS [18].

### Isothermal titration calorimetry

ITC measurements were performed using a MicroCal iTC200 microcalorimeter (GE Healthcare) calibrated according to the manufacturer′s instructions. NcImp*α* and the SV40 NLS peptide were prepared and dialyzed in buffer (20 mM Tris-HCl, pH 8.0, and 100 mM NaCl). The sample cell was loaded with 50 *μ*M NcImp*α* that was titrated with the SV40 NLS peptide at a concentration of 1 mM (protein:peptide molar ratio of 1:20). Titrations were conducted at 10°C and consisted of 20 injections of 2.0 *μ*L in an interval of 240 s with a 1000 rpm homogenization speed. The heat of dilution was determined by titration of the peptide sample into the protein sample buffer (20mM Tris-HCl, pH 8.0, and 100 mM NaCl) in separate control assays and was subtracted from the corresponding titrations. The assays temperature was chosen to avoid protein aggregation displayed for higher temperatures and to permit direct comparison to previously Imp*α*/SV40NLS ITC studies performed in the same condition [19]. The data were processed using MicroCal Origin Software to obtain values for stoichiometry (N), dissociation constants (*K*_*d*_), enthalpy (ΔH), and binding-type input parameters were adjusted to obtain the best fitting model. The values of *K*_*d*_ and ΔH were used to calculate free energy (ΔG) and entropy (ΔS) values.

### Crystallization, X-ray data collection and structure determination

NcImp*α* was concentrated to 12 mg/ml using an Amicon 30 kDa cutoff filter unit (Millipore) and stored at -20°C. Crystals of NcImp*α* in complex with SV40 NLS were obtained at a 1:8 protein:peptide molar ratio in 20 mM Bicine, pH 8.5, and 20% (w/v) polyethylene glycol 6000 at 4°C [20] using MRC2 Well Crystallization Plates and an Orix4 system (Douglas Instruments).

X-ray diffraction data were collected from a single crystal of NcImp*α*/SV40NLS at a wavelength of 1.0 Å using a synchrotron radiation source (X25 beamline, National Synchrotron Light Source, NSLS, Upton, NY, USA) and a PILATUS detector. A crystal was mounted in a nylon loop and flash-cooled in a stream of nitrogen at -173.15°C using 20% (v/v) glycerol as the cryoprotectant. The crystal-to-detector distance was 270 mm with an oscillation range of 0.5°, resulting in the collection of a total of 720 images. The data were processed using the HKL2000 suite [21]. The crystal belonged to the space group *P*2_1_2_1_2_1_ (Table 1) and was isomorphous to previously obtained crystals [20].

**Table 1:** X-ray data-collection and refinement statistics for NcImp*α*/SV40NLS structure.

*Values in parentheses are for the highest resolution shell*.	
*Diffraction data statistics*	
Unit cell parameters (Å)	a = 45.1; b = 64.4; c = 185.6
Space group	P2_1_2_1_2_1_
Unique reflections	54993 (5195)^a^
Completeness (%)	99.6 (95.8)^a^
Mosaicity	0.40
Multiplicity	12.4 (9.3)^a^
R_*merge*_ † (%)	6.8 (84.3)^a^
Average I/*σ*(I)	37.5 (2.0)^a^
*Refinement statistics*	
Resolution (Å)	36.98–1.75 (1.81–1.75)^a^
R_*cryst*_ ‡ (%)	18.3 (28.5)^a^
R_*free*_ § (%)	21.0 (30.2)^a^
Number of non-H atoms	
Protein	3164
Peptide	113
Solvent	349
Ramachandran plot ††	
Residues in most favored regions(%)	99
Residues in disallowed regions (%)	0.23
Mean B-factor(Å^2^)^*e*^	36.7

† $R_{merge}=\frac{\sum_{hkl}(\sum_{i}(|I_{hkl,i}-<I_{hkl}>|))}{\sum_{hkl,i}<I_{hkl}>}$, where *I*_*i*_ (hkl) is the intensity of an individual measurement of the reflection with Miller indices hkl and hI(hkl)i is the mean intensity of this reflection. Calculated for I > -3*σ* (I) [21].

‡ $R_{cryst}=\frac{\sum_{hkl}(||Fobs_{hkl}|-|Fcalc_{hkl}|)}{|Fobs_{hkl}|}$, where ∣*Fobs*∣ and ∣*Fcalc*∣ are the observed and calculated structure-factor amplitudes, respectively.

§ *R*_*free*_ is equivalent to *R*_*cryst*_ but was calculated with reflections (5%) omitted from the refinement process. Calculated based on the Luzzati plot with the program SFCHECK [31].

† † Calculated with the program PROCHECK [31].

^*a*^Values in parentheses are for the highest-resolution shell.

NcImp*α*/SV40NLS crystal structure was determined by molecular replacement using the program Phaser [22] and the coordinates of Imp*α* from *Mus musculus* in complex with the nucleoplasmin NLS (PDB ID: 3UL1, chain B; [15]) as the search model. Rounds of manual modeling were performed using the program Coot [23] and the crystallographic refinement (positional and restrained isotropic individual B factors with an overall anisotropic temperature factor and bulk-solvent correction) was performed using the program phenix.refine [24], considering free R factors. Structure quality was evaluated using the program MolProbity [25], and interactions were analyzed using the program LIGPLOT [26]. Figures were generated using the program PyMOL [27]. Superposition of Imp*α* structures was performed using LSQ algorithm [28] present in the software package CCP4 [29].

### PDB accession code

Coordinates and structure factors from NcImp*α*/SV40NLS have been deposited in the PDB under accession code 4RXH.

## Results

### Structure of NcImp*α* in complex with SV40NLS

NcImp*α* was expressed as an N-terminal truncation lacking residues 1–74 (which corresponds to the IBB domain; [30]) that are responsible for autoinhibition. Furthermore, crystallization was performed in the presence of an NLS ligand to stabilize the truncated protein, as previously reported [17, 20], and to investigate the protein-NLS binding features.

X-ray diffraction data collection and refinement statistics of the NcImp*α*/SV40 NLS complex are summarized in Table 1. The crystals are not isomorphous to other Imp*α*/SV40 complexes [10] and diffracted to high resolution (1.75Å), which is the highest resolution obtained for an Imp*α* to date.

**Fig 1:**
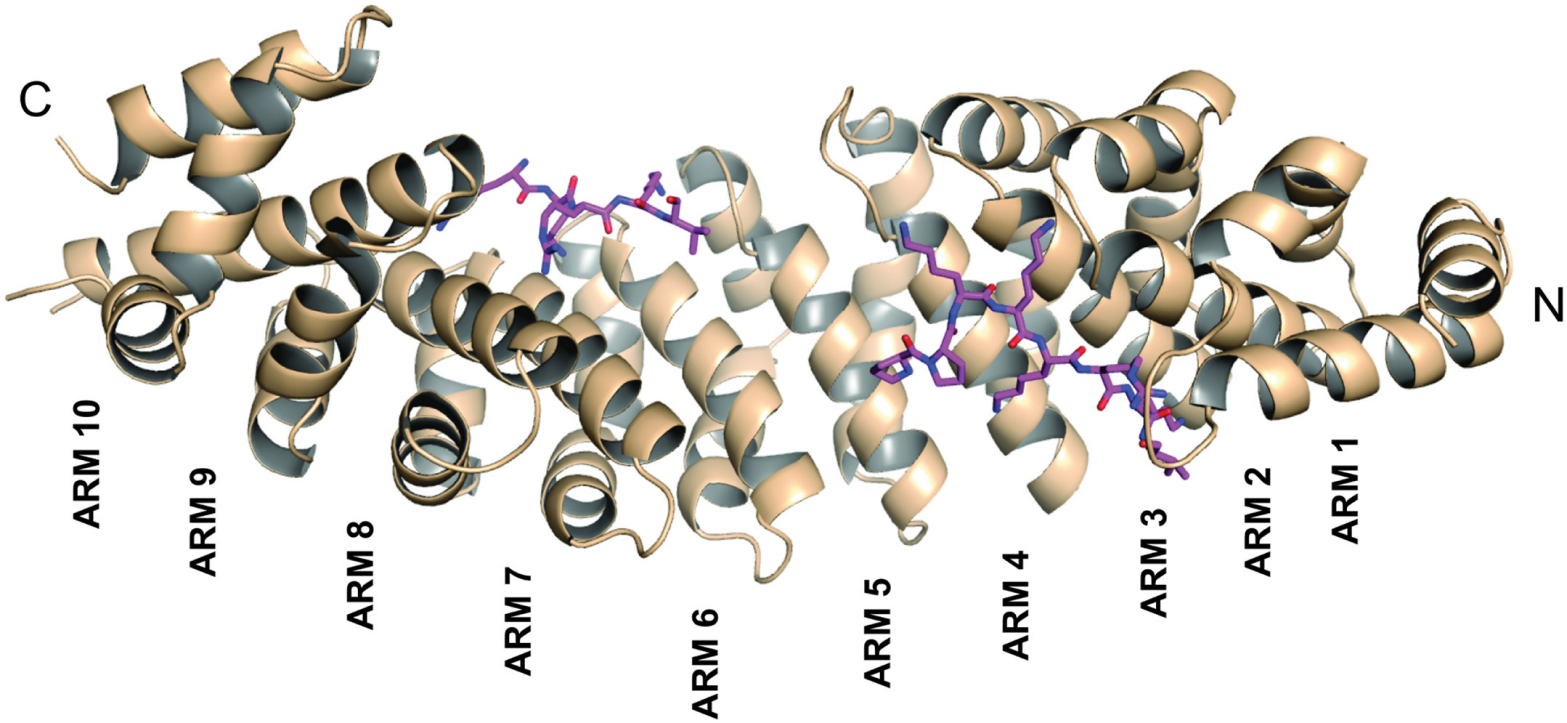
Overall structure of the NcImp*α* in complex with the SV40 NLS peptide. NcImp*α* structure represented in cartoon exhibits an elongated and curved shape composed of ten tandem Arm repeats. The SV40NLS peptides, represented in magenta sticks, are bound to the major (Arms 1–4) and minor (Arms 4–8) binding sites of NcImp*α*.

The final model of the NcImp*α*/SV40NLS complex consists of 428 residues of NcImp*α* (79–507), the peptide ligands bound to the major (seven residues) and minor (four residues) sites, and 349 water molecules (Table 1). The structure exhibits an elongated and curved shape composed of ten tandem Arm repeats, each containing three *α*-helices (H1, H2 and H3;), as observed in other Imp*α* structures [9, 10, 16, 30, 32] (Fig 1). The loop containing residues 461–465 could not be modeled due an absence of electron density. The concave surface of the protein maintains the conserved array of Trp and Asn residues and negatively charged residues that interact with positively charged residues from the NLS ligands.

Superposition of the C*α* atoms of NcImp*α* (residues 83–505) with other importin-*α* structures results in a root-mean-square deviation (r.m.s.d.) of 1.78 Å for Imp*α* from *M. musculus* (MmImp*α*; 3UL1; [33]), 1.77 Å for human Imp*α*5 (HsImp*α*5; 4WV6; [34]), 1.22 Å for human Imp*α*1 (HsImp*α*1; 2JDQ; [35]), 1.21 Å for rice Imp*α* (OsImp*α*; [36]) and 1.13 Å for yeast Imp*α* (ScImp*α*; [9]).

### Binding of the SV40 NLS peptide at the NcImp*α* major binding site

**Fig 2:**
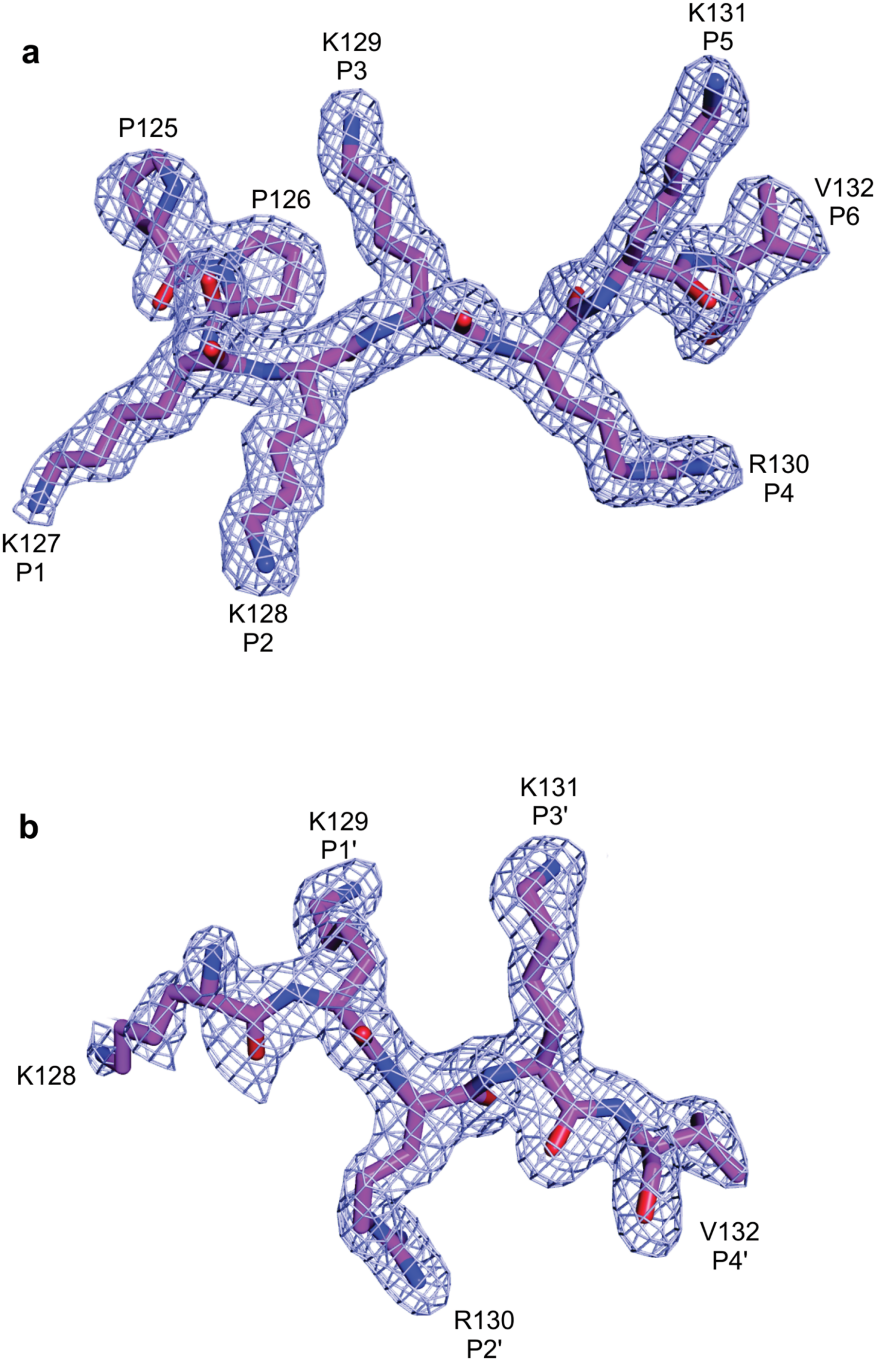
Electron-density map (coefficients 2∣*F*_*obs*_∣ − ∣*F*_*calc*_∣) of the NcImp*α*/SV40NLS structure in the area corresponding to SV40NLS peptides (contoured at 2.0 s.d.) at major (A) and minor (B) binding sites.

The SV40 NLS peptide binds the major binding site of NcImp*α* in an extended conformation with an orientation that is antiparallel to the Arm repeats. The electron density for the peptide in the major binding site is well defined, allowing for the unambiguous modeling of the eight peptide residues (^125^ *PPKKKRKV* ^132^, Fig 2a). The SV40 NLS peptide exhibits a conserved binding mode at the major binding site, which is analogous to mouse (MmImp*α*; [10]), yeast (ScImp*α*; [9]) and rice (OsImp*α*; [16]) Imp*α* proteins. The N-terminal residue (P125) also exhibits a conformation that is similar to that observed in the structures of OsImp*α*/SV40 NLS and MmImp*α* in complex with an extended SV40 NLS peptide (G110-G132, referred as CN-SV40NLS) [37]. ScImp*α*/SV40 NLS and MmImp*α*/SV40 NLS were crystallized using truncated version of the SV40 NLS peptide (P126-V132).

**Fig 3:**
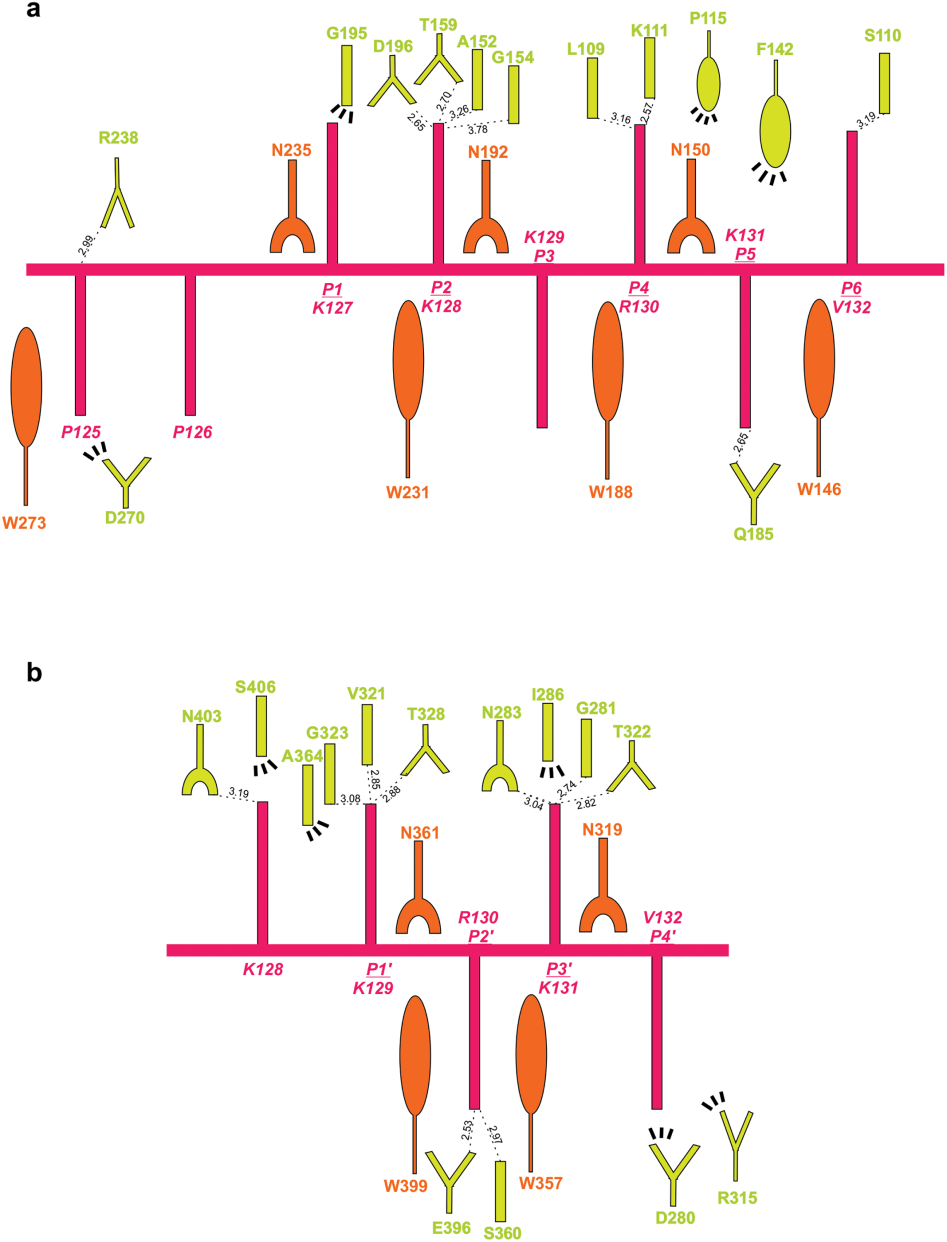
Schematic diagram of the interactions between SV40NLS and NcImp*α* for major (A) and minor (B) binding sites. The SV40NLS peptide main and side-chains are drawn in magenta. NcImp*α* residues interacting with the peptide are indicated by yellow (side-chain) and orange (main-chain). Polar contacts are shown as dashed lines and arcs with radiating spokes indicate hydrophobic contacts.

The average B-factor of the peptide at the major binding site (33.2 Å^2^) is lower than the average B-factor for the protein (37.7 Å^2^), indicating stability for the interaction of the peptide at this site. The conserved asparagines N150, N192 and N235 of NcImp*α* stabilize the backbone of the SV40 NLS peptide via hydrogen bonds, whereas the residues W146, W188 and W231 form pockets for the side chains of K129 and K131 at the *P*_3_ and *P*_5_ positions of the SV40NLS peptide, respectively. The K127 of SV40NLS participates in hydrophobic interactions with G195 of NcImp*α* at the *P*_1_ binding site. K128 of SV40 NLS forms hydrogen bonds with G154, A152 and T159 and a salt bridge with D196 of NcImp*α*, as observed for lysine residues occupying the *P*_2_ position in previous structures [9, 10]. In the *P*_4_ site, the side chain of R130 interacts with L109 and K111 of NcImp*α* via hydrogen bonds and hydrophobic interactions with P115 and S153. Finally, K131 (position *P*_5_) forms hydrogen bonds with Q185 and hydrophobic interactions with F142, and V132 (position *P*_6_) forms a hydrogen bond with S110 of NcImp*α* (Fig 3a, S1 Fig).

Interestingly, the N-terminal residue P125 of the SV40NLS peptide, that is not in the *P*_1_ − *P*_6_ positions, forms hydrogen bonds and hydrophobic interactions with R238 and D270 of NcImp*α*. P125 aids in the stabilization of interactions between the protein and a peptide NLS at the major binding site, as observed previously for MmImp*α*/CN-SV40NLS and OsImp*α*/SV40NLS structures [16, 37].

### Binding of the SV40 NLS peptide at the NcImp*α* minor binding site

The electron density for the SV40 NLS peptide at the minor binding site is also well defined and allows the unambiguous modeling of four residues of the peptide (^128^ *KRKV* ^132^, Fig 2b). As observed for the major binding site, asparagines residues (N319 and N361 in this case) of NcImp*α* define and guide the backbone of the peptide. The average B-factor for the SV40 NLS peptide bound at the minor binding site of NcImp*α* is higher than the average B-factor for the protein (39.3 and 37.7 Å^2^, respectively), indicating a lower stability for the peptide at this site compared with the major site.

The interactions between the SV40NLS peptide and NcImp*α* in the minor site exhibit greater similarities with the interactions between this peptide and OsImp*α* [16](Table 2) than those with MmImp*α* or ScImp*α* [9, 10]. In the NcImp*α*/SV40NLS structure, K129 at the *P*_1_’ position forms hydrogen bonds with G323, V321 and T328 of NcImp*α*. R130, which occupies the *P*_2_’ position, is accommodated between the hydrophobic side chains of W357 and W399 and interacts with E396 via salt bridges and with S360 via hydrogen bonds, which results in the lowest B-factor value (36.2 Å^2^) compared to the other residues of the peptide. K131 at the *P*_3_’ position is stabilized by helix dipoles, negatively charged residues and hydrogen bonds with N283, G281, and T322. Finally, V132 at *P*_4_’ interacts with D280, R315 and N319 of NcImp*α* via hydrophobic interactions (Fig 3b, S2 Fig).

**Table 2:** Binding to specific pockets of Imp*α*/NLS structures from different organisms.

								Minor Site							Linker						Major Site						
Imp*α*/NLS*								$P_{1}^{\prime }$	$P_{2}^{\prime }$	$P_{3}^{\prime }$	$P_{4}^{\prime }$										*P*_1_	*P*_2_	*P*_3_	*P*_4_	*P*_5_	*P*_6_	
NcImp*α*/SV40				P	P	K	K	**K**	**R**	**K**	**V**								P	P	**K**	**K**	**K**	**R**	**K**	V	
OsImp*α*/SV40				P	P	K	K	**K**	**R**	**K**	**V**	G	G					S	P	P	**K**	**K**	**K**	**R**	**K**	V	G
MmImp*α*/SV40						P	K	**K**	**K**	**R**	**K**	V								P	**K**	**K**	**K**	**R**	**K**	V	
ScImp*α*/SV40					S	P	K	**K**	**K**	**R**	**K**								S	P	**K**	**K**	**K**	**R**	**K**	V	
MmImp*α*/CN–SV40							**K**	**K**	**R**	**K**	**V**						…	**A**	**P**	**P**	**K**	**K**	**K**	**R**	**K**	**V**	
MmImp*α*/hPLSCR4					**G**	**S**	**I**	**I**	**R**	**K**	**W**	**N**															
MmImp*α*/TPX2							G	**K**	**R**	**K**	**H**	G	P	S	P	V	**K**	**M**	**I**	**K**	L						
MmImp*α*/Nucleoplasmin								**K**	**R**	**P**	**A**	**A**	**T**	K	**K**	**A**	**G**	**Q**	**G**	**A**	**K**	**K**	**K**	**K**			
MmImp*α*/N1N2				C	G	R	K	**K**	**R**	**K**	**T**	**E**	**E**	**E**	**S**	**P**	**L**	**K**	**D**	**K**	**A**	**K**	**K**	**S**	**K**	G	Y
MmImp*α*/RB						C	G	**K**	**R**	**S**	**A**	**E**	**G**	**S**	**N**	**P**	**P**	**K**	**P**		**L**	**K**	**K**	**L**	**R**	G	Y
MmImp*α*/FEN1					S	**S**	**A**	**K**	**R**	**K**	**E**	**P**	**E**	**P**	**K**	**G**	**S**		**T**		**K**	**K**	**K**	**A**	**K**	**T**	
MmImp*α*/Bimax1		R	R	R	R	**P**	**R**	**K**	**R**	**P**	**L**	**E**	**W**	**D**	**E**	**D**	**E**	**E**	**P**	**P**	**R**	**K**	**R**	**K**	**R**	**L**	**W**
MmImp*α*/Bimax2		R	R	R	**R**	**R**	**R**	**K**	**R**	**K**	**R**	**E**	**W**	**D**	**D**	**D**	**D**	**D**	**P**	**P**	**K**	**K**	**R**	**R**	**R**	**L**	**D**
OsImp*α*/A89	V	H	K	**T**	**V**	**L**	**G**	**K**	**K**	**K**	Y	M															
OsImp*α*/B54				**S**	**V**	**L**	**G**	**K**	**R**	**K**	**R**	**H**	P	K	V												
ScImp*α*/c–Myc					P	A	A	**K**	**R**	**V**	**K**	L	D						**P**	**A**	**A**	**K**	**R**	**V**	**K**	**L**	**D**
HsImp*α*5/Nup50		**P**	**L**	**G**	**S**	**M**	**A**	**K**	**R**	**N**	**A**	**E**	**K**	**E**	**L**	**T**	**D**	…									

* Binding to specific binding pockets of Imp*α* based on structural data are shown in bold. The NLSs are aligned as observed to bind to the NLS-binding sites ($P_{1}^{\prime }$ –$P_{4}^{\prime }$, minor binding site; *P*_1_—*P*_6_, major binding site, as defined in [11]). The suspension points corresponds to the extension of the NLS peptides that not bind into the linker region. NcImp*α*/SV40NLS; OsImp*α*/SV40NLS [16]; MmImp*α*/SV40NLS [10]; ScImp*α*/SV40NLS [9];MmImp*α*/CN-SV40NLS [37]; MmImp*α*/hPLSCR4 [38]; MmImp*α*/TPX2 [39]; MmImp*α*/Nucleoplasmin [10]; MmImp*α*/N1N2 [7]; MmImp*α*/RB [7]; MmImp*α*/FEN1 [40]; MmImp*α*/Bimax1 [41]; MmImp*α*/Bimax2 [41]; OsImp*α*/A89 [16]; OsImp*α*/B54 [16]; ScImp*α*/c-Myc [11]; HsImp*α*5/Nup50 [42].

### Affinity of the SV40 NLS peptide for NcImp*α*

**Fig 4:**
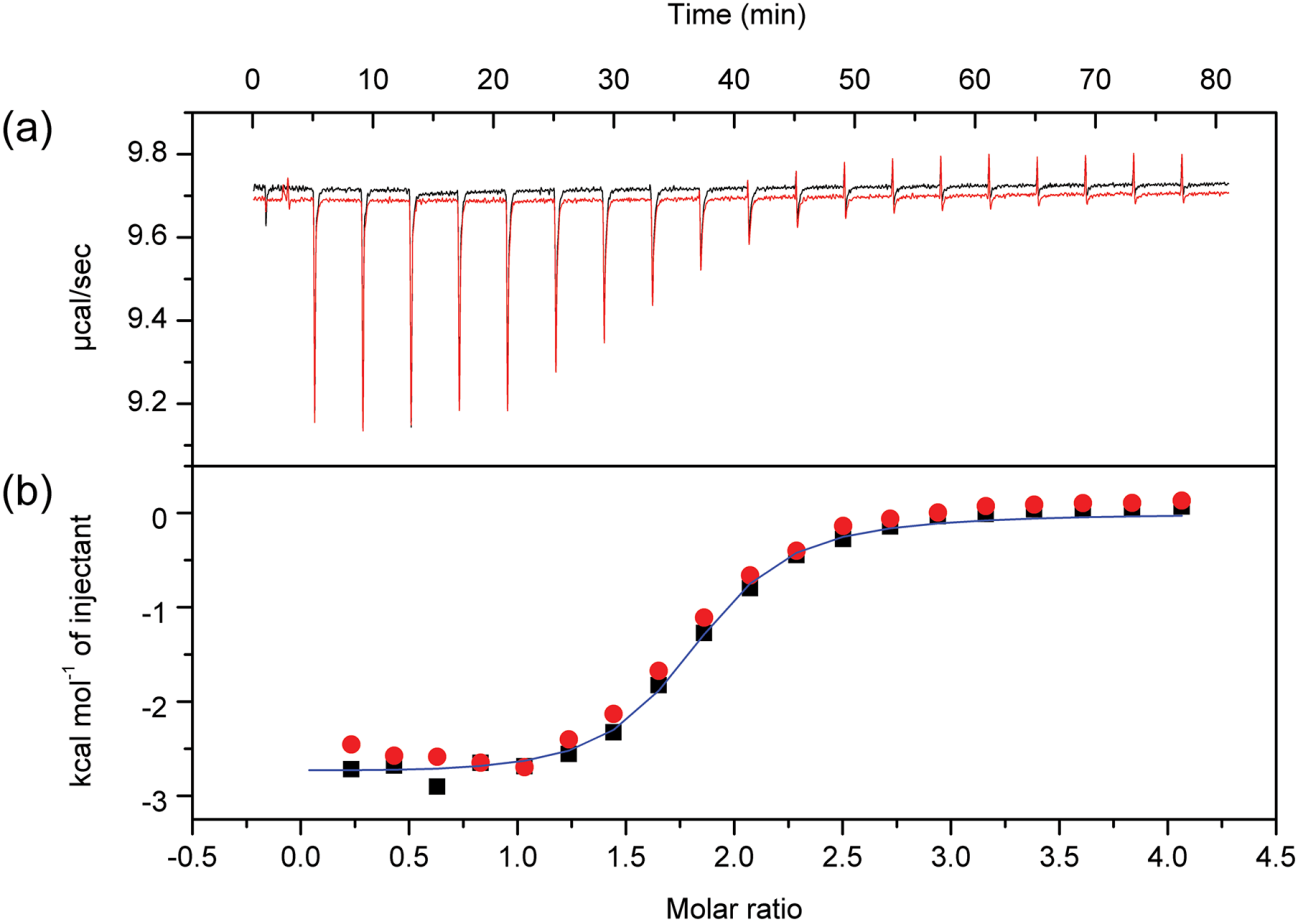
Isothermal calorimetric data for SV40NLS peptide binding to NcImp*α*. **(a)** Raw power output (*μ*cal/s) per unit time (min) of replicate titrations **(b)** Integrated data (kcal.mol^−1^ of injectant versus molar ratio of SV40NLS to NcImp*α*). These data were obtained from the raw power output as the area underneath each peak, which is then corrected for baseline heat injections and SV40NLS dilution heat and mixing. The solid line represents the best fit of the data.

The affinity and other thermodynamic parameters for the association of NcImp*α* and the SV40 NLS peptide were evaluated using ITC. A protein:peptide molar ratio of 1:20 was sufficient to yield a sigmoidal titration curve representing an exothermic process during complex formation (Fig 4). A two non-symmetrical binding site interaction model was selected based on the determined structure, which indicates the presence of two NLS binding sites in the Imp*α* protein with different binding modes. The equilibrium dissociation constants of *K*_*d*_ = 1.23±0.22 *μ*M and *K*_*d*_ = 1.69±0.46 *μ*M (Table 3) indicated that the SV40NLS peptide may bind to both sites but with higher affinity for one site. These results are consistent with previous structural and functional results that indicate the presence of the major and minor binding sites in Imp*α* in complex with the SV40NLS peptide [9, 10] and are comparable with ITC experiments performed with MmImp*α*/SV40NLS complex [19]. Furthermore, the negative contribution for the enthalpy (ΔH) and positive value for the entropy (ΔS) suggest that both hydrogen bonds and hydrophobic interactions play a role in this interaction, whereas conformational changes are unfavorable. These data corroborate with the structural information obtained in the present study.

**Table 3:** Isothermal titration calorimetry data for SV40NLS binding to NcImp*α*.

	Major site	Minor Site
N	0.89 ± 0.02	0.86 ± 0.02
K_*d*_ (*μ*M)	1.23 ± 0.22	1.69 ± 0.46
ΔH (kcal/mol)	-2.47 ± 0.06	-3.21 ± 0.12
ΔS (cal/mol/deg)	18.3	15.1

## Discussion

### Comparison of NcImp*α* with other Imp*α* structures

**Fig 5:**
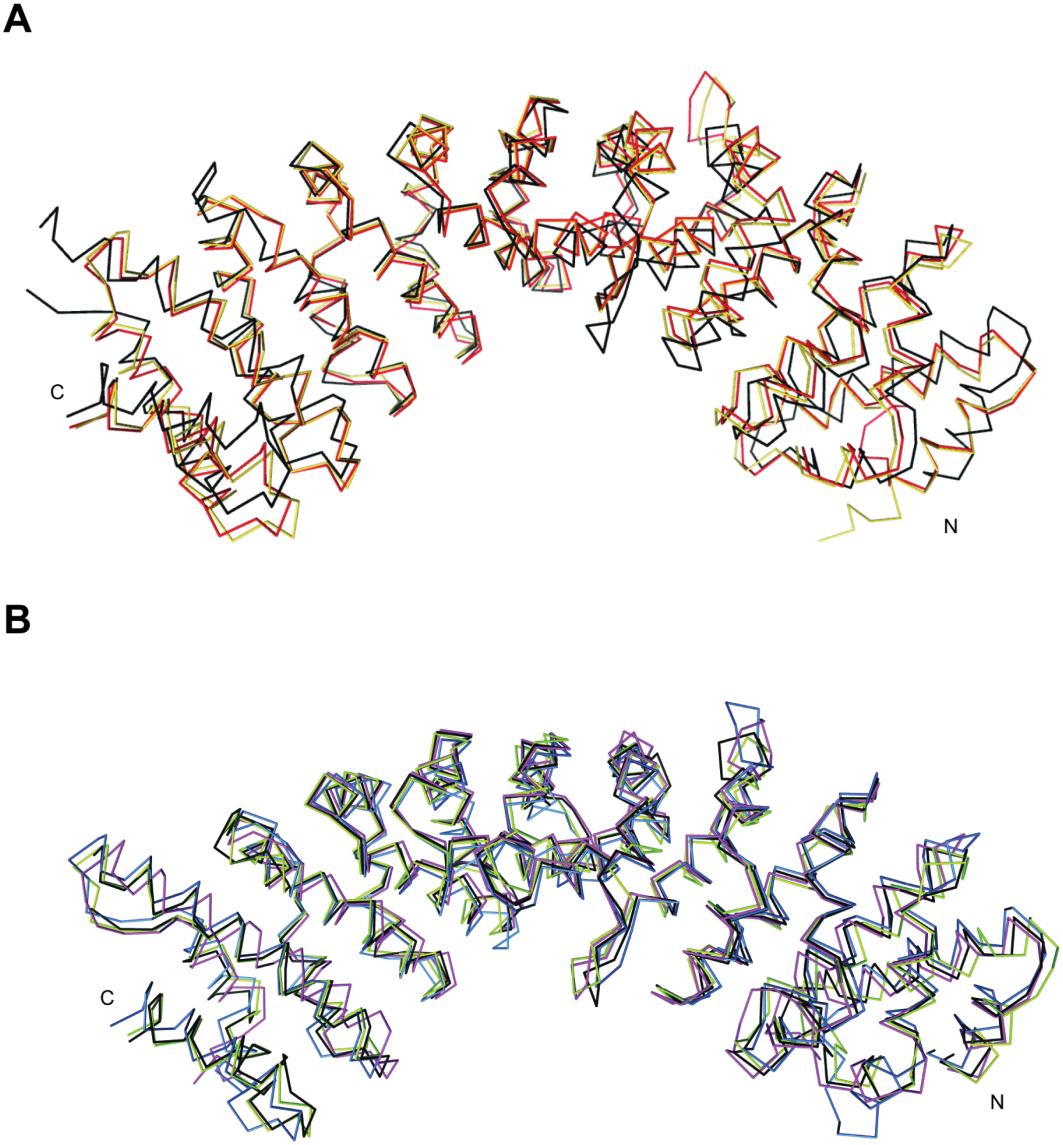
Comparison among Imp*α* crystal structures from different organisms. The Imp*α* proteins were superimposed using the C*α* atoms of each protein (residues 83 -505) **(a)** NcImp*α*, MmImp*α* (PDB ID: 3UL1) and HsImp*α*1 (PDB ID: 4WV6) are represented in black, red and orange, respectively. **(b)** NcImp*α*, OsImp*α* (PDB ID: 4BQK), ScImp*α* (PDB ID: 1BK5) and HsImp*α*5 (PDB ID: 2JDQ) are represented in black, blue, green and pink, respectively.

The crystal structure of NcImp*α* resembles other Imp*α* structures [9, 10, 16] but exhibits a conformation that is more concave compared with MmImp*α* (Fig 5a). Its superposition (residues 83–504) with other Imp*α* proteins reveals a higher r.m.s.d. for C*α* atoms for MmImp*α* (residues 78–496; PDB ID: 3UL1; r.m.s.d.: 1.78 Å) and HsImp*α*1 (residues 78–493; PDB ID: 4WV6; r.m.s.d.: 1.77 Å) (Fig 5a) compared with ScImp*α* (residues 89–507; PDB ID: 1BK5; r.m.s.d.: 1.13 Å), OsImp*α* (residues 74–493; PDB ID: 4BQK; r.m.s.d.: 1.21 Å) and HsImp*α*5 (residues 84–502; PDB ID: 2JDQ; r.m.s.d.: 1.22 Å) (Fig 5b). The primary sequence identities between NcImp*α* and other Imp*α* proteins directly reflect the r.m.s.d. values for C*α* atoms: ScImp*α* (64.69% identity; r.m.s.d.: 1.13 Å), OsImp*α* (63.16% identity; r.m.s.d.: 1.21 Å), HsImp*α*5 (60.22% identity; r.m.s.d.: 1.22 Å), MmImp*α* (46.26% identity; r.m.s.d.: 1.78 Å), HsImp*α*1 (45.01%; r.m.s.d.: 1.77 Å). Furthermore, ScImp*α*, OsImp*α* and HsImp*α*5 belong to the same phylogenetic family as NcImp*α* (*α*1 family; [43, 44]), whereas MmImp*α* and HsImp*α*1 belongs to a different family (*α*2 family). These sequence and structural comparisons raise an important question concerning Imp*α* from different organisms or isoforms from the same organism. How different is the binding mode of an NLS ligand to a particular Imp*α*?

Several studies have shown that Imp*α* proteins from different families exhibit preferences for specific NLSs [45–48]. Thus, examining the binding mode of the SV40NLS peptide, which has been crystallized with various Imp*α* proteins, may provide insights into the binding specificities of these proteins. Crystal structures of Imp*α*/SV40NLS complexes have been determined for ScImp*α* [9], MmImp*α* (in complex with SV40NLS and with an extended SV40NLS peptide, referred to as CN-SV40— [10, 37]), OsImp*α* [16] and NcImp*α* (the present study). In all of these structures, the SV40NLS peptide binds strongly to the major site via several interactions, resulting in well-defined electron density and B-factor values that are similar to or lower than the average B-factor value for the protein. These results are consistent with the determined affinities for the MmImp*α*/SV40NLS [19] and NcImp*α*/SV40NLS complexes using ITC, in which both studies demonstrated the presence of two binding sites for the peptide exhibiting with different affinities. In all of these structures, SV40NLS binding to the major site is essentially identical, in which all six positions (*P*_1_ − *P*_6_) of the peptide (^127^ *KKKRKV* ^132^, Table 2) are occupying similar regions at the protein. The main differences are related to the number of peptide residues bound before the *P*_1_ position (OsImp*α* exhibits 4 residues before *P*_1_, NcImp*α* exhibits 2 residues, MmImp*α* exhibits 1 residue and MmImp*α*/CN-SV40NLS exhibits 4 residues). However, these differences result from the length of the SV40NLS peptide used in these structural studies rather than differences in Imp*α* proteins from different organisms.

### The role of the minor binding site for NcImp*α* specificity

The major site was usually associated with the binding of monopartite NLSs, whereas the minor site was associated with a secondary binding site for bipartite NLSs [7, 10, 40]. Recently, studies have demonstrated that some monopartite NLSs may bind to only the minor site [16, 38], indicating the importance of this binding site for Imp*α* specificity for cargo proteins.

**Fig 6:**
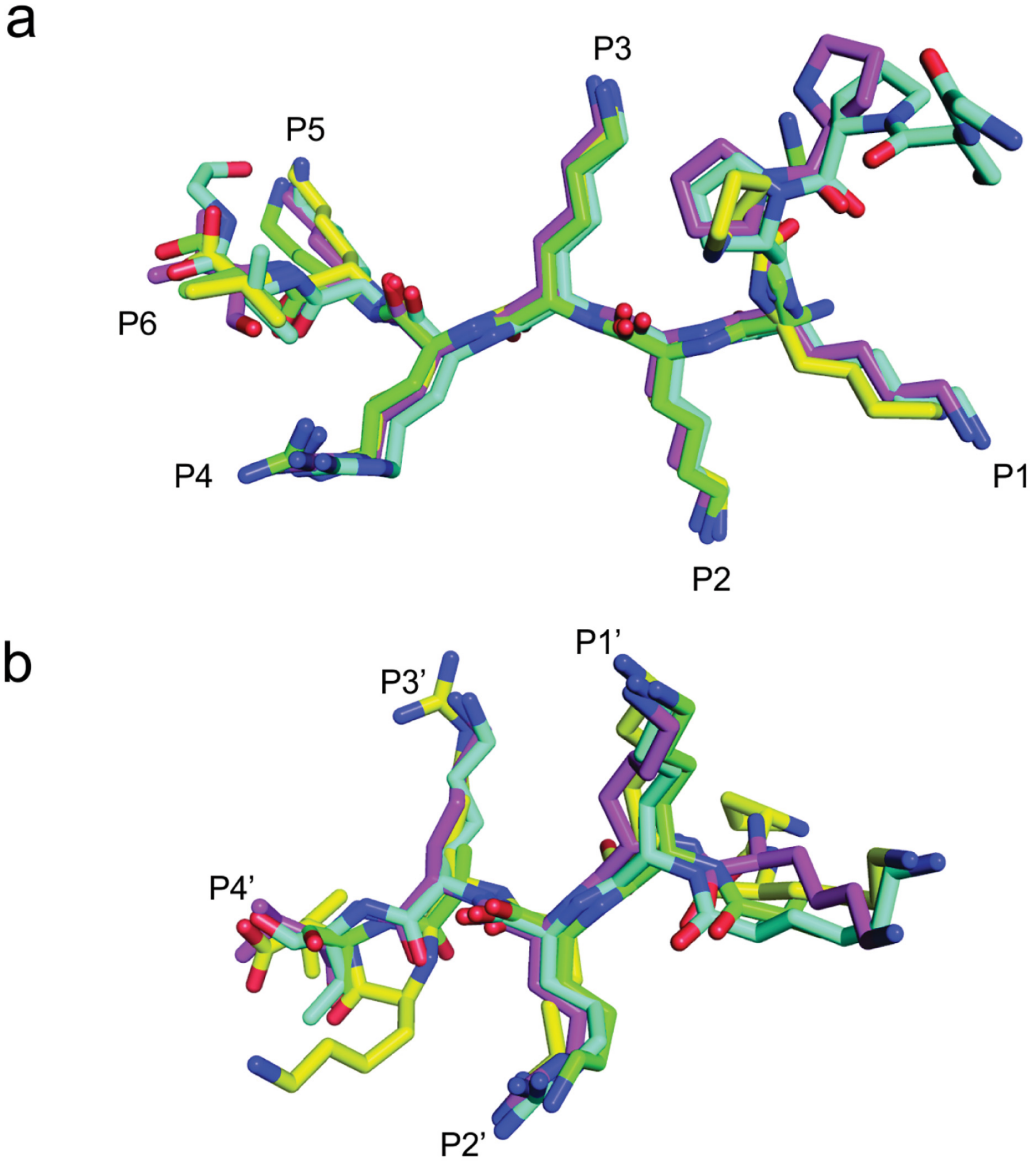
Comparison of SV40NLS peptides binding to the major (a) and minor (b) binding sites for Imp*α* structures from different organisms. SV40NLS peptides in complex with NcImp*α* (magenta), ScImp*α* (green), OsImp*α* (blue) and MmImp*α* (yellow) were superimposed using the C*α* atoms of the peptides. Positions binding corresponding to the major (P_1_-P_5_) and minor (P_1_′-P_4_′) sites are identified along the chains (binding pocket labels were based on peptide binding with NcImp*α*).

In contrast to the binding of SV40NLS to the major site (Fig 6a), the binding mode of the peptide at the minor binding site is different among different Imp*α* proteins. As shown in Table 2 and Fig 6b, SV40NLS binds to ScImp*α* and MmImp*α* via Lys and Arg residues at the *P*_1_’ and *P*_2_’ positions, whereas the peptide binds OsImp*α* and NcImp*α* via Lys residues at both *P*_1_’ and *P*_2_’ positions, i.e., the binding of the peptide is shifted one position in the OsImp*α* and NcImp*α* structures.

Interestingly, Lys and Arg residues at the *P*_1_’ and *P*_2_’ positions, respectively, are observed for the majority of monopartite (hPLSCR1, hPLSCR4, c-Myc, and TPX2 [11, 38, 39, 49]) and bipartite NLS complexes (nucleoplasmin, N1N2, RB and FEN1 [10, 37, 40]), and it is also the predominant binding mode for MmImp*α*/CN-SV40NLS [37]. Furthermore, the authors of MmImp*α*/SV40NLS structure [10] also observed staggering of peptide one position N-terminally which may lead to an alternative binding mode with Lys and Arg residues at the *P*_1_’ and *P*_2_’ positions. The binding of KK residues at the *P*_1_’ and *P*_2_’ positions for SV40NLS in MmImp*α* and ScImp*α* seems to be exceptions in these particular cases.

An additional interesting characteristic of SV40NLS binding to Imp*α* is the presence of additional interactions between both the N- and the C-termini of the peptide and MmImp*α* in the minor site: two positions before *P*_1_’ (P126, N-terminus) and in the *P*_5_’ position (V132). These interactions are observed in only the mammalian receptor despite all SV40NLS peptides used for crystallization with Imp*α* proteins have these same residues. Structural studies on OsImp*α* have indicated that the plant-specific NLS peptide preferentially binds at the minor site in OsImp*α* and at the major site in MmImp*α* [16]. The similarity of SV40NLS binding at the minor site between OsImp*α* and NcImp*α* raises questions concerning the importance of this NLS binding site for the import mechanism of *N. crassa*.

**Fig 7:**
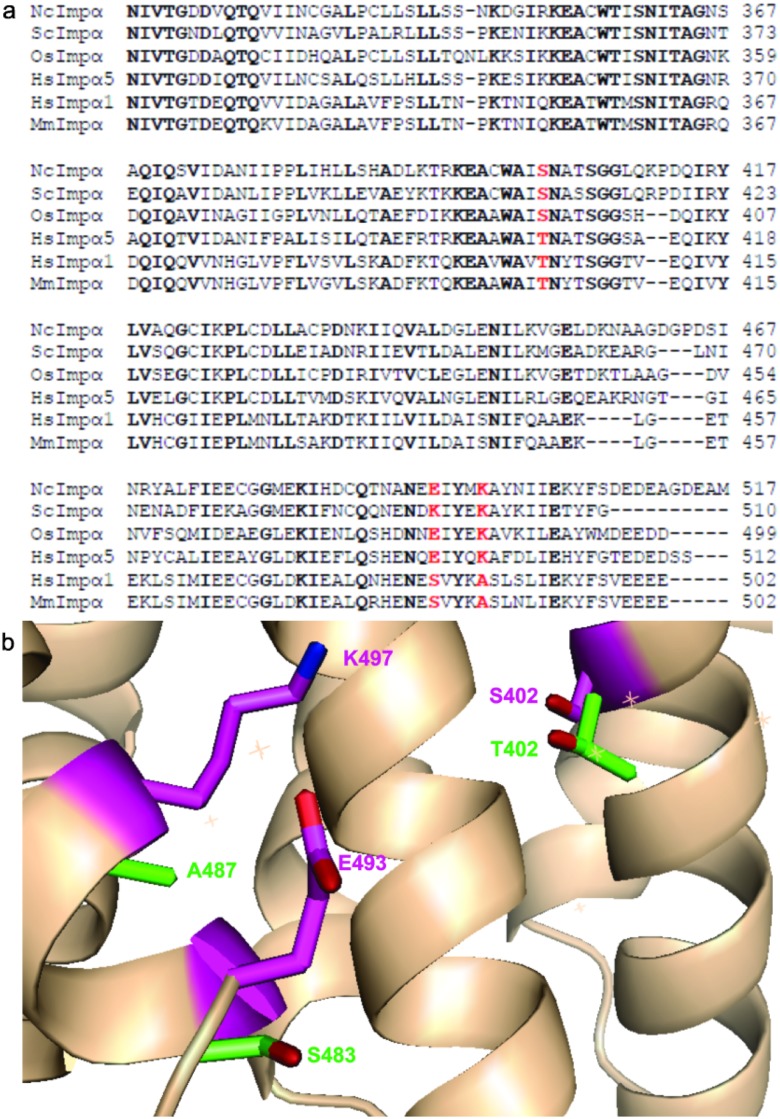
Non-conserved residues between Imp*α* proteins near to the minor NLS binding region. (a)Partial alignment of amino acid sequences of Imp*α* proteins from different organisms. Conserved residues are shown in black. The residues S402, K497 and E493 for NcImp*α* and their equivalent residues for Imp*α* proteins are shown in red. **(b)** Superposition between C*α* of NcImp*α* and MmImp*α* structures (performed as described in Fig 5) highlights the residues S402, K497 and E493 of NcImp*α* (magenta) and the residues T402, S483 and A487 of MmImp*α* (green). These particular residues may be associated to NLS binding specificity to the minor NLS binding site. E493 side-chain is shown in a hypothetical conformation in this figure because this residue presents lack of electron density in this structure due to its high flexibility.

Chang and colleagues [16] observed the presence of non-conserved residues between OsImp*α* and MmImp*α* (S394, R427, E434, E480, and K484 for OsImp*α*) in the region near the minor site (Arm repeats 8 and 9) and toward the C-terminus may be responsible for the specificity of NLS binding for OsImp*α*. Multiple sequence alignment of Imp*α* proteins (Fig 7a) indicates that some of these residues that are present in OsImp*α* (S394, E480 and K484) are also present in NcImp*α* (S402, E493, and K497) and in other Imp*α* proteins from the *α*1 family (S408, R443 and K500 in ScImp*α* and E491 and K495 in HsImp*α*). Structural comparison between NcImp*α* and MmImp*α* (Fig 7b) shows that these three residues (S402, E493, and K497 in NcImp*α*) are substituted by T402, S483 and A487 for MmImp*α* indicating that favorable interaction of particular peptides may occur. Particularly, T402 in MmImp*α* (S402 in NcImp*α*) has been reported to lie in an identical position as S394 in the OsImp*α* structure, sterically preventing the binding of the plant-specific NLS peptide to MmImp*α* [16]. However, these substitutions may also prevent the binding of K127 of SV40NLS to NcImp*α* and OsImp*α*, explaining the presence of this interaction in MmImp*α*, which belongs to the *α*2 family, and the absence of this interaction in NcImp*α*, which belongs to the *α*1 family.

The majority of monopartite (hPLSCR4, c-Myc, and TPX2 [11, 38, 39]) and bipartite NLS complexes (nucleoplasmin, N1N2, RB and FEN1 [7, 10, 40]) presented KR residues at the $P_{1}^{\prime }$ and $P_{2}^{\prime }$ positions which seems to be the most favorable residues to occupy these positions. The binding of KK residues atthe $P_{1}^{\prime }$ and $P_{2}^{\prime }$ positions for SV40NLS in MmImp*α* seems to be an exception which probably occurs because it is also favorable [10] to bind additional residues at the N- and C-terminal regions of the peptide for some Imp*α* proteins. For OsImp*α*/SV40NLS and NcImp*α*/SV40NLS complexes, KR binding at the $P_{1}^{\prime }$ and $P_{2}^{\prime }$ positions is more favorable because fewer interactions occur with the N- and C-terminal regions of the peptide. This observation accounts for the shift in the binding of SV40NLS to OsImp*α* and NcImp*α* compared with MmImp*α*.

Affinity assays between NLS peptides and Imp*α* proteins have been performed based in indirect affinity measurements [16, 40, 49–54], using Surface Plasmon Resonance (SPR) [32], and ITC [19, 38, 55]. Indirect affinity measurements between NLSs and Imp*α* proteins reported dissociation constants in the nM range and permitted the comparison among these molecules. SPR experiments was only able to estimate the peptide-protein affinity in the *μ*M range which was lately confirmed by ITC experiments. The comparison between ITC analysis of MmImp*α*/SV40NLS [19] and NcImp*α*/SV40NLS is consistent with the features of the minor binding site observed in the structure. The higher affinity of the peptide at the minor binding site for MmImp*α* (*K*_*d*_ = 0.98±0.08 *μ*M) compared with NcImp*α* (*K*_*d*_ = 1.69±0.46 *μ*M) may be associated with the presence of additional interactions at N- and C-termini in the MmImp*α*/SV40NLS complex. The crystal structure of this complex [10] has revealed that the residues K131 and V132 of the SV40 NLS peptide form salt bridges with the conserved E354 and R315 of MmImp*α*, respectively. Additionally, a hydrogen bond between V132 and R135 aids in the stabilization of the backbone of the SV40 NLS C-terminus. The importance of additional interactions N- and C-termini has been also observed with ITC assays for a phosphorylated NLS peptide which enhanced by 10-fold its affinity to MmImp*α* compared to unphosphorylated version [54]. Furthermore, it has been observed that the full length nucleoplasmin protein binds to Imp*α*/Imp*β* complex with a 2-fold increase in affinity compared to just nucleoplamin NLS peptide [53]. Interesting, ITC studies with the hPLSCR4 NLS peptide (^273^ *GSIIRKWN* ^280^) which binds only the minor site of MmImp*α* [38] showed lower affinity (*K*_*d*_ = 48.7±6.5 *μ*M) compared with that of the SV40NLS peptide. This fact may be mainly attributed to the absence of a positively charged residue in the hPLSCR4 peptide at the *P*_1_’ position.

In conclusion, despite the structural similarities among Imp*α* proteins, this study and other recent studies with this receptor from different organisms or different isoforms from the same organism clearly demonstrated differences in the binding specificities for cargo proteins. The differences between NcImp*α* and MmImp*α* may result from the phylogenetic distance among the proteins and the functions of each protein family in organism development, which results in differences in affinities for NLSs. The elucidation of NcImp*α* in complex with specific NLSs peptides from fungi may provide an explanation for the differences between these proteins.

## Supporting Information

S1 Fig Schematic diagram of the interactions between the SV40NLS peptide (purple) and NcImp*α* (orange) at the major binding site. Polar contacts are shown with dashed lines, and hydrophobic contacts are indicated bay arcs with radiating spokes. Carbon, nitrogen and oxygen atoms are shown in black, blue and red, respectively. Generated with the program LIGPLOT [26].
(EPS)

S2 Fig Schematic diagram of the interactions between the SV40NLS peptide (purple) and NcImp*α* (orange) at the minor binding site. Polar contacts are shown with dashed lines, and hydrophobic contacts are indicated bay arcs with radiating spokes. Carbon, nitrogen and oxygen atoms are shown in black, blue and red, respectively. Generated with the program LIGPLOT [26].
(EPS)
